# The Effects of *Berberis Vulgaris *Juice on Insulin Indices in Women with Benign Breast Disease: A Randomized Controlled Clinical Trial

**Published:** 2018

**Authors:** Sanaz Asemani, Vahid Montazeri, Behzad Baradaran, Mohammad Amin Tabatabiefar, Saeed Pirouzpanah

**Affiliations:** a *Nutrition Research Center, Tabriz University of Medical Sciences, Tabriz, Iran. *; b *Department of Thoracic Surgery, Faculty of Medicine, Tabriz University of Medical Sciences\ Surgery Ward, Nour-Nejat Hospital, Tabriz, Iran. *; c *Immunology Research Center, Tabriz University of Medical Sciences, Tabriz, Iran. *; d *Department of Genetics and Molecular Biology, School of Medicine, Isfahan University of Medical Sciences, Isfahan, Iran. *; e *Department of Biochemistry and Dietetics, Faculty of Nutrition and Food Sciences, Tabriz University of Medical Sciences, Tabriz, Iran.*

**Keywords:** Benign breast disease, *Berberis vulgaris*, Homeostasis model assessment, Breast cancer, Fasting blood sugar

## Abstract

The aim of this study was to investigate the effect of *Berberis vulgaris *(*BV*) juice consumption on insulin homeostasis, glycemic profiles of patients with benign breast disease (BBD). This parallel design, triple-blind, randomized and placebo controlled clinical trial was conducted on 85 eligible women diagnosed with BBD who recruited from Nour-Nejat hospital, Tabriz, Iran. Participants were randomly allocated into either intervention group who received *BV* juice (480 mL/day, n = 44) or *BV* juice placebo at the same time (480 mL/day, n = 41). After a 7 day run-in period, treatments were administered for the duration of 8 weeks. Participants, care givers and those who assessed laboratory analyses were blinded to the assignments (IRCT registry no: IRCT2012110511335N2).

The relative treatment effects of *BV *supplementation showed decreased serum levels of insulin for 19%, C-peptide for 8%, homeostasis model assessment of insulin resistance index (HOMA-IR) for 16% and glucose to insulin ratio for 22% but HOMA-B increased 44% relative to placebo group over 8 weeks *BV* supplementation. Although these changes were not statistical significant, the mean changes for C-peptide and HOMA-B were significant just after adjusting for baseline data and covariates. Administration of *BV *juice showed controlling effects on HOMA related indices, consequently might have beneficial effects on insulin signaling-related functions in women with benign breast tumor.

## Introduction

The increasing prevalence of benign breast neoplasia incidence is a public health concern in different populations (1, 2). Several lines of evidence from epidemiologic studies have given rise to the notion that environment risk factors are important in the development of breast malignancy (3). 

Benign breast disease (BBD) is a well-established variable predispose women to breast cancer (BC) possibly later in life (4). The BBD consists of different histological subtypes classified as non-proliferative lesions, proliferative lesions without atypia, and atypical hyperplasia (5). Studies have shown that women with proliferative lesions of BBD are susceptible to develop malignant pathogenesis during their life time (1.5 times higher risk), whereas women with atypical hyperplastic lesions are subject to 4 times increased risk of BC (1, 3). 

Among various risk factors identified in the etiology of BBD, less information exists regarding environment and diet-related factors on BBD development (6). Recently, it has been well-addressed that insulin is a tumor promoting growth factor (7). Connecting peptide or C-peptide, a marker of individual’s own insulin secretion with a long half-life, is considered to be one of the key risk factors of a later onset BC and remarked as a sensitive marker to validate the variables related to insulin levels (8). Among limited number of studies, Horner and coworkers indicated that dietary intakes of low fat and high fiber could be useful in the prevention of breast nodularity (9), suggesting that insulin metabolism could be nominated as a possible explanatory mechanism (10). To the best of our knowledge, insulin-dependent biomarkers have not been investigated in the contribution of dietary factors among patients afflicted with benign breast lesions.

Barberry* or Berberis vulgaris *(*BV*) belongs to the plant family *berberidaceae* (11). Its numerous biological properties have been mostly related to the high content of berberine (isoquinolinealkaloid) in *BV* fruit and root (12). Recent studies have shown that *BV* or berberine exerts therapeutic effects on some clinical manifestations of insulin resistant (IR)-dependent disorders such as diabetes (13-15). Since, the effects of *BV *consumption on insulin-dependent metabolic indices regarding to tumor promoting features have not been addressed thus far. Therefore, this randomized, triple-blind, and placebo-controlled clinical trial was conducted to investigate the effects of *BV* juice consumption on insulin-related indices among women with BBD.

## Experimental

*Materials and Methods*

*Study subjects*

**Figure 1:**
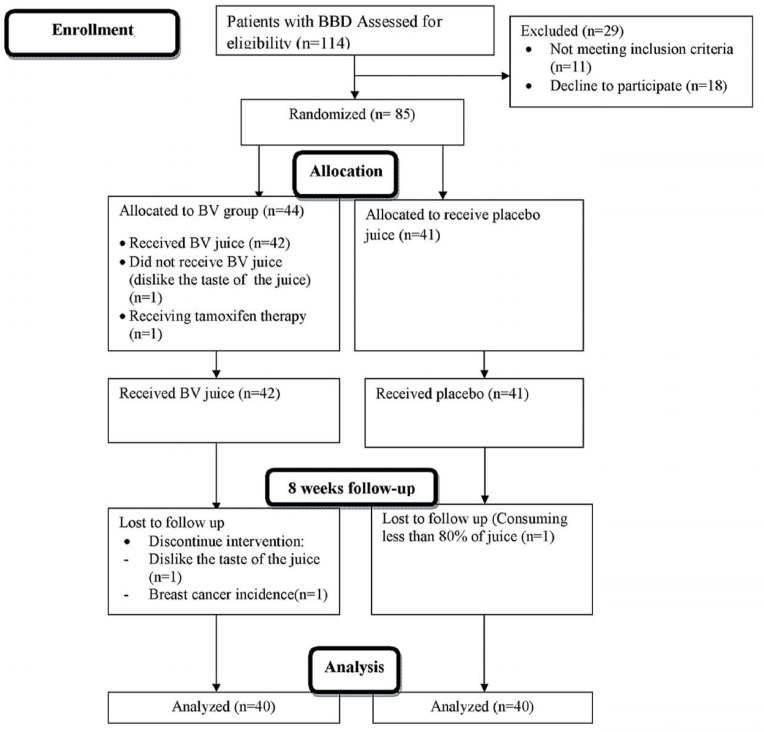
CONSORT flow diagram of the progress through different time compartments of a parallel randomized trial of *BV *and *BV *placebo received groups during 8 weeks of intervention. BBD, breast benign tumor disorder; *BV, Berberis vulgaris*

This parallel design, triple-blind, randomized and placebo controlled clinical trial was conducted on premenopausal women (19-52 years old) with benign breast tumor, whose disease was diagnosed by ultra-sonography imaging results, recruited from Nour-Nejat private hospital, Tabriz, Eastern Azerbaijan, between July 2013 and October 2014. The BBT patients did not have any surgical procedure or mastectomy prior to inclusion in the trial. Eligibility criteria included: being diagnosed with fibrocystic changes (n = 69), fibroadenoma (n = 11), written and oral informed consent, being at the interval of at least 2 years from diagnosis of BBD and no former history of malignancy in any part of the body. We excluded patients with smoking habits in previous years (ever and former smokers); pregnancy and lactating statue during the treatment; afflicted with acute and chronic illness including: cardiovascular disease, renal or liver malfunctions; other malignancies; hyperthyroidism and other hormone-related disorder (type 1 diabetes, adrenal gland disorders, polycystic ovary syndrome and hypoglycemia); gastrointestinal inflammatory illnesses (peptic ulcer, gastritis and inflammatory bowel syndrome); history of other benign lesions; any sign of bleeding and trauma reported by patients or physician; intolerance to the content of *BV *juice; consumption of medications like anticoagulants (such as aspirin); omega-3 (> 2000 mg/d); glucocorticoids; methotrexate; alpha-tocopherol(> 400 IU/d) supplements, epilepsy-related drugs and any medical history related to chemo-, radio-, and hormone-therapy. The sample size was calculated based on information outlined by Gu *et al. *(16) and estimated as 30 patients with BBT at each group, but after considering the attenuated power of analysis for stratified random sequence generation, 30% further enrolment was planned in the study protocol (Figure 1). At last, 85 women diagnosed with BBD were included and randomly assigned into two groups via stratified random allocation. All participants received written informed consent form prior to enrolment, which is completed by each participant. The study was conducted due to the revised guidelines released in the Declaration of Helsinki. The study design was approved by Ethics Committee Center at Tabriz University of Medical Sciences (Ethics no: 9233). IRCT registration number is IRCT2012110511335N2.

*Study design*

The selected eligible participants were asked to follow a 7 days run-in period prior to the beginning of the interventions. Run-in period was performed in order to obtain a situation to reduce the possible interaction of other dietary resources containing isoquinoline alkaloid to reach a better adherence in 8 weeks. Generally, participants was also asked not to change their common life style, particularly their usual (i.e., habitual) diet. At first interview of run-in period, dietitian instructed the participants to fill 3 days collection of 24-h dietary record (two weekdays and one on a nearest weekend), by describing portion size of food servings through the food model guideline. A dietitian carried out questioning through a face-to-face interview. The eligible participants were randomly assigned to either intervention or placebo group. Randomization was applied using a list taken out from computer-generated randomization software.

All subjects were randomized to receive 8-weeks treatment with 480 mL doses of either *BV* juice or placebo daily both in lunch and dinner meals (a glass in each meal equal to 240 mL). Intervention for the duration of 8 weeks was planned for this trial. All tetra pack *BV* juice (240 mL) was prepared by Takdaneh Agro-Industrial Company (Takdaneh, Co. Ltd., Marand, East-Azerbayjan, Iran). Each serving of BV juice contained carbohydrates: 55 gr/500 mL; protein: 600 mg/500 mL; fat: 10 mg/500mL; calcium: 50 mg/500 mL; sodium: 100 mg/500 ML and vitamin C: 80 mg/500 mL.

**Table1:** Demographic and dietary characteristics of BBD patients in placebo and intervention groups at the baseline of study

**Characteristics**	**Placebo (n = 40)**		**Intervention (n = 40)**		***P-*** **value** *
	**Mean ± S.D.**	**Median**	**Mean ± S.D.**	**Median**	
**Demographic data**					
Age at diagnosis (years)	38.45 ± 6.9	40.0	36.17 ± 7.6	40.0	0.16
Age at firs delivery (years)	21.4 ± 3.9	20.5	21.0 ± 3.8	19.5	0.66
Age at first menses (years)	13.4 ± 1.6	13.0	13.0 ± 1.5	13.0	0.75
**Dietary intake data**					
Total calorie intake (kcal/day)	10.26 ± 4.07	9.07	11.99 ± 6.19	10.68	0.49
Protein intake (g/day)	52.49 ± 19.20	47.24	56.41 ± 22.49	51.49	0.41
Carbohydrate intake (g/day)	1359 ± 457	1298	1407 ± 483	1272	0.21
Fat intake (g/day)	5.10 ± 3.05	4.60	4.28 ± 1.93	4.20	0.25
Saturated fatty acids(g/day)	32.89 ± 12.20	32.49	37.15 ± 13.96	35.05	0.04
Mono saturated fatty acids(g/day)	9.61 ± 4.19	9.18	10.65 ± 5.09	9.70	0.16
Poly saturated fatty acids (g/day)	10.47 ± 9.52	10.24	11.20 ± 10.34	10.36	0.25
Dietary fiber (g/day)	0.83 ± 1.16	0.76	0.69 ± 0.91	0.41	0.13
Soluble fiber (g/day)	113 ± 182	76	120 ± 99	84	0.64
Insoluble fiber (g/day)	13.31 ± 6.73	11.74	14.93 ± 5.66	13.48	0.61

* Independent sample t-test was used to compare data. All data were expressed as mean±standard deviation (S.D.) and related median.

**Table 2:** Characteristics of histopathological, familial and multivitamin use status among BBD patients in placebo (n = 40) and intervention (n = 40) groups at the baseline of study

**Characteristics**	**Intervention(n = 40)**	**Placebo(n = 40)**	***p-*** **value** *
**Histopathological characteristics**			
Fibrocystic	34(85.0) **	35(87.5)	< 0.001
Fibroadenoma	6(15.0)	5(12.5)	
Others			
**Family history of BC**			
No	35(87.5)	30(75.0)	< 0.001
Positive	5(12.5)	10(25.0)	
**Family history of BBD**			
No	40(100.0)	39(97.5)	< 0.001
Positive	0	1(2.5)	
**Smoker**			
Never	40(100)	40(100)	N/A
Ever	0	0	
Current	0	0	
**Multivitamin use**			
No	38(95.0)	35(87.5)	< 0.001
Yes	2(5.0)	5(12.5)	
**Duration of multivitamin use (months)**			
≤2	1(50.0)	3(60.0)	0.680
>2	1(50.0)	2(40.0)	
**Vitamin E capsule use**			
No	22(55)	25(62.5)	0.11
Yes	18(45)	15(37.5)	
**Omega3 capsule use**			
No	33(82.5)	28(70)	< 0.001
Yes	7(17.5)	12(30)	

BC, breast cancer; BBD, benign breast disorders.

* Chi-square test was performed.

**  Data was expressed in the form of number of participants (relative frequency, %).

**Table 3:** Serum levels of glycaemic and insulinemic biomarkers at baseline compartment of study and 8 weeks after the intervention in women with BBT who received *BV* supplementation (*BV* group) versus placebo juice consumers (control).

**Variable**	**Baseline (n = 40)**				**8-weeks follow-up (n = 40)**				**Absolute treatment effect**			**Relative treatment effect**
	**n**	**Mean**	**S.D.**	***P***	**n**	**Mean**	**S.D.**	***P***	**Mean**	**95%CI**	***P*** c	
**Insulin (U/mL)**												
Control	39	0.38	0.33	N/A	39	0.50^*^	0.17	N/A	N/A	N/A	N/A	1.00
*BV*	35	0.49	0.43	0.09	35	0.51^*^	0.22	0.72	0.10	(-0.08-0.28)	0.26	0.81
**C-peptide (ng/mL)**												
Control	40	2.27	1.06	N/A	40	2.97^*^	1.13	N/A	N/A	N/A	N/A	1.00
*BV*	40	2.56	1.12	0.23	40	3.42^*^	1.44	0.16	-0.15	(-0.62-0.32)	0.13	0.92
**FBS (mg/dL) ** ^d^												
Control	40	92.22	14.73	N/A	40	87.85	12.14	N/A	N/A	N/A	N/A	1.00
*BV*	40	89.25	9.33	0.28	40	91.00	10.85	0.22	-6.12	(-12.7-0.50)	0.97	1.06
**HOMA-IR** e												
Control	39	0.09	0.09	N/A	40	0.11	0.04	N/A	N/A	N/A	N/A	1.00
*BV*	39	0.10	0.09	0.34	37	0.11	0.06	0.19	0.01	(-0.03-0.06)	0.44	0.84
**HOMB ** f												
Control	39	13.30	49.87	N/A	39	9.69^*^	6.54	N/A	N/A	N/A	N/A	1.00
*BV*	36	10.26	15.86	0.05	36	9.80^*^	9.49	0.05	375	(21-730)	0.86	1.44
**QUIKI ** g												
Control	39	0.72	0.15	N/A	40	0.62^*^	0.06	N/A	N/A	N/A	N/A	1.00
*BV*	38	0.67	0.13	0.15	37	0.62^*^	0.07	0.94	-0.04	(-0.11-0.02)	0.29	0.94
**G:I ratio ** h												
Control	39	345.08	219	N/A	39	186.01^*^	49.14	N/A	N/A	N/A	N/A	1.00
*BV*	36	271	194	0.11	36	185.23^*^	79.07	0.92	0.15	(-0.05-0.35)	0.28	0.78

All data are expressed in geometric mean ± S.D.. N/A: not applied to this model.

a Paired t-test was performed to compare within changes in intervention group during the study.

b  Independent sample t-test was performed between group.

c  Repeated measure of ANOVA was carried out in the main effect of model.

e  Homeostatic model assessment.

f  Assessment of B-cell functionality.

g  quantitative insulin sensitivity check index glucose to insulin ratio.

h Glucose to insulin ratio.

**Table 4:** Changes of dependent variables (plasma levels of glycemic and insulinemic biomarkers) with *BV* supplementation in women with BBD

**Variable**	**Placebo group**	***BV*** ** group**	***P-*** **value ** b
**Insulin (U/mL)**			
Model 1^c^	0.54 ± 0.02^a^	0.55 ± 0.02	0.783
Model 2 d	0.54 ± 0.02	0.55 ± 0.2	0.669
**C-peptide (ng/mL)**			
Model 1	2.97 ± 0.07	3.33 ± 0.12	0.029
Model 2	2.96 ± 0.07	3.35 ± 0.13	0.018
**FBS (mg/dL)**			
Model 1	87.64 ± 0.40	94.70 ± 1.24	< 0.001
Model 2	87.70 ± 0.41	94.20 ± 1.44	< 0.001
**HOMA-IR ** e			
Model 1	0.13 ± 0.01	0.14 ± 0.01	0.183
Model 2	0.13 ± 0.01	0.13 ± 0.01	0.633
**HOMB ** f			
Model 1	666 ± 78	973 ± 147	0.114
Model 2	664 ± 76	977 ± 142	0.048
**QUIKI ** g			
Model 1	0.66 ± 0.01	0.65 ± 0.01	0.447
Model 2	0.66 ± 0.01	0.65 ± 0.01	0.359
**G:I ratio ** h			
Model 1	0.62 ± 0.02	0.63 ± 0.02	0.697
Model 2	0.62 ± 0.01	0.63 ± 0.01	0.252

a All data are expressed in geometric mean ± S.E..

b  This *p-value* obtained from performing ANCOVA.

c  Weighted least square was performed in ANOVA with adjustment for baseline variable (model 1).

d  Model 2 obtained from performing ANCOVA after adjustment for the frequency of lactation (n) and BMI (kg/m2) at baseline in addition to baseline variable.

e  Homeostatic model assessment.

f  Assessment of B-cell functionality.

g  quantitative insulin sensitivity check index.

h  Glucose to insulin ratio.

**Figure 2.**
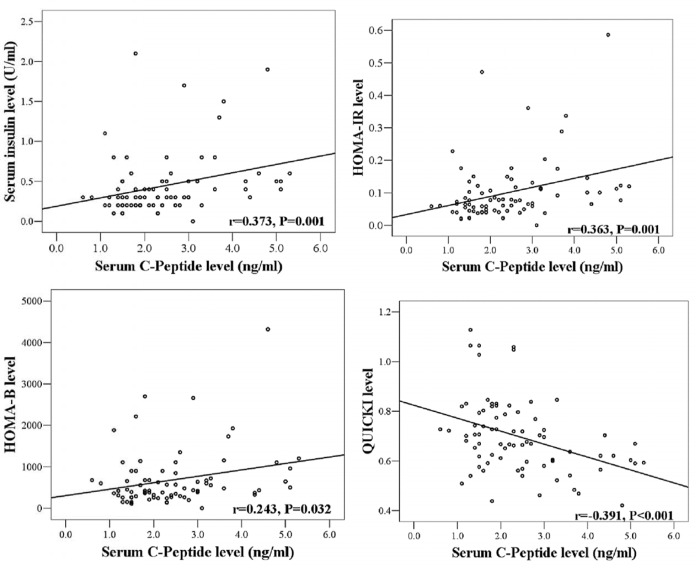
Pearson correlation coefficient value obtained to show the validity of insulin-related indices (dependent variable) in association with serum C-peptide concentration (independent variable) at baseline compartment of the study (n = 80

Spectrophotometric method measured absorbance of berberine at 418 nm in *BV* juice (17). After measuring five different samples of *BV* juices, the average berberine content was determined 2.03 ± 0.03 μM/mL. The company declares no addition of any preservatives in *BV* juice tetra-pack.

Weekly checklist of *BV* consumption was handed in to the eligible patients for assessing the probable intolerance to interventions, detailing undefined events, doubt or accidental consumption, and other medical events. If weighted amount of remaining juice in bottle was more than 20% of total amount of juice, the subject was considered to be none respondent to the intervention. In addition, bottles were collected in every two weeks. During the intervention, data on 3-day 24 h dietary and physical activity records were collected once in every two weeks. Physical examinations at these visits were done by physician in order to determine any clinical changes during the study, including malignancy, diabetes, hepatic complications and pregnancy after enrolment and during the interventions, which were considered as exclusion criteria. Intervention follow up continued until December 2014.

Participants in placebo group consumed 480 mL of placebo juice had a content which was normalized to become identical in calorie, vitamin content, taste, size and color to the *BV* juice. To meet the allocation concealment criteria, white opaque plastic was used to cover juice pack similarly. Juice packs were provided in every two weeks (18). Subjects were asked to store juices in refrigerator at 4ºC. Sequence generation and allocation concealment were listed and marked by designer of study and implemented by clinic personnel who were unaware of allocation at the time of enrolment. Participants, clinic care givers who had been responsible to hand in plastic bags and also laboratory personnel were blinded. The average of nutrients intake level for each participant obtained from 24 h dietary records at baseline were analyzed by Nutritionist IV software (version 3.5.2; 1994, N-Squared Computing, San Bruno, CA). To assess the concurrence of dietary data, a validated food frequency questionnaire with 136 food items (19) was used.

*Biochemical analyses*

Serum samples of 80 patients were collected by considering overnight 12 h fasting. Blood sampling process had not being performed in duration of 3^rd^ till 5^th^ days of menstruation (follicular phase) both at baseline and after the 8-week follow-up period. Blood were transferred centrifuged (Gallenkamp centrifuge) at 3000 × g at 20 °C for 10 minutes to separate serum supernatant. Sera samples transferred into 1.5mL microtubes and stored at -70 °C until sample analysis. Outcomes of the study consist of serum levels of C-peptide, fasting blood sugar (FBS) and insulin. They were assessed using a specific enzyme linked immunosorbent assay (ELISA) kits for fasting blood sugar (FBS) (Pars-Azmoon, Cat. No: 5825; Tehran, Iran), insulin by Monobind (Cat No: 5825-300; California, USA), and C-peptide using Monobind kit (Cat No: 2725-300; California, USA). The internal coefficient variations of biomarkers were reported about 92%. The measurements were carried out following instruction provided by manufacturer. The HOMA related indices were calculated using the HOMA-IR= [FBS (mg/dL) × insulin (IU)/405] and HOMA-B= [(360 × insulin (IU)]/ [(FBS (mg/dL)-63] × 100. The quantitative insulin sensitivity check index (QUICKI) was estimated using 1/[(logarithm of insulin+ logarithm of FBS)]. To attenuate systematic errors for each biomarker, measures were done at the same time in a laboratory run and random order. A numeric label code was used instead of each patient’s name, to attain blinding at laboratory analysis. 

*Statistical analysis*

Statistical analysis was carried out using SPSS software package (version 13.0; SPSS Inc.). Paired samples t-test was used to compare mean values of a variable from baseline to the endpoint of study within each group. Independent sample t-test was used to compare a variable between *BV* and placebo groups at certain time point. The mean change in the variable from baseline to the 8 weeks of follow-up between the two arms of intervention was calculated by absolute treatment effect and tested by a repeated-measures linear mixed model. The relative effect is a term used to define probability of an outcome in one treatment group relative to that in the placebo group and likewise. The odds ratio (OR) can interpret the proportional change in the treatment group relative to placebo group. Dependent variable was adjusted for baseline data and estimated through a weighted least squares (WLS) regression model. Subsequently, linear mixed model was used to consider covariates including frequency of lactation (n), and body mass index (BMI, kg/m^2^) at baseline.* P* values less than 0.05 has been reported as statistically significant.

## Results

*Baseline characteristics*

The excluded subjects during the enrollment or during the 8-week follow-up are depicted in the flow chart diagram (Figure 1). None of the study subjects were in the follicular phase of their menstrual cycle, and all were in pre-menopausal status. The average rate of being respondent to interventions was 94.3% in case of using *BV* juice and 90.7% in placebo group. The *BV* and its placebo juice did not lead to any adverse effects in patients till the trial was completed. The baseline characteristics of participants were not significantly different between the intervention and placebo groups. 

The habitual dietary components and lifestyle-related factors of all subjects did not differ significantly at the length of intervention (Table 1). Among study subjects, pathological classification of benign disease identified to be 85.0% with fibrocystic and 15.0% with fibroadenoma in the *BV* group. Similarly, 87.5% of participants in placebo group had fibrocystic tumor significantly different from the relative frequency of those subjects with fibroadenoma (12.5%, *P* < 0.001) (Table 2).

***The primary outcomes***

 Serum levels of C-peptide correlated significantly with serum insulin levels (*r =* 0.37, *P* < 0.01) and HOMA-IR values (*r *= 0.36, P < 0.01). C-peptide was inversely correlated with the variable of QUICKI (r = - 0.39, *P* < 0.001) (Figure 2). 

 Although comparing the results of the 8-weeks intervention with baseline (Table 3) showed significant increases in the average levels of insulin at both groups, insulin decreased by 19% after 8 weeks intervention in *BV* group relative to placebo [relative treatment effect = 0.81, (*P* = 0.26)]. There was also a significant increase in C-peptide levels both in the placebo, and *BV* groups (Table 3). Serum C-peptide decreased by 8% after treatment with* BV* relative to placebo, whereas these changes were not statistically significant (Table 3). HOMA-IR showed 16% decrease in *BV* group relative to the placebo, but it was not statistically significant (Table 3). Although there were significant decreases of HOMA-B levels in both groups (*P* < 0.001), HOMA-B increased by 44% relative to placebo after 8 weeks treatment of the *BV* group (Table 3). Mean changes of QUICKI level was higher in placebo group while compared to *BV* group. Glucose to insulin (G: I) ratio decreased 22% in the *BV* group, which is comparable to the relative change observed in placebo over the 8 weeks (Table 3). Table 4 showed values adjusted for baseline variable in model 1 and subsequently further controls for potently related covariates (i.e., the frequency of lactation (n), and BMI at baseline) undertook in model 2. Results indicated that there was no significant increase in insulin level in both models (Table 4). The mean levels of C-peptide or FBS after performing adjustments for corresponding baseline values, increased significantly (*P* < 0.05). HOMA-IR level increased in model 1, but it did not reach the statically significant level (0.13 ± 0.01 to 0.14 ± 0.01, *P* = 0.18). The model 2 analysis showed a remarkable increase in HOMA-B level in post-adjustment model controlled for the frequency of lactation (n), and BMI at baseline through WLS model (Table 4).

## Discussion

 There is growing body of evidence showing that obesity in association with IR seems to be a causative factor for developing BC pathogenesis (4). Our findings over the present randomized controlled trial could support the assumption that *BV* juice might reduce HOMA-IR and G:I ratio, and in the meantime, enhance the level of HOMA-B. 

 The advantage of using berberine in relation to enhance insulin sensitivity in terms of HOMA-B resulted in the study of Lu and coworkers (20). It was also supported by our finding showed that *BV* intervention increased HOMA-B by 44% relative to placebo in WLS model. 

 However, to the best of our knowledge thus far, this is the first study investigated the effect of *BV *on insulin-related indices in benign breast disease. A clinical trial on T2DM patients for 3 months performed by Gu and colleagues showed that the daily supplementation of berberine can lead to a significant decrease in fasting and post-load glucose levels as well as glycated hemoglobin (HbA1c) (16). Consistent to our findings in BBD patients, Zhang* et al. *(21) reported that daily supplementation of berberine for 3 months caused significant reductions in fasting and post loaded plasma levels of glucose in T2DM patients with dyslipidemia. Affuso* et al*. (22) also suggested that the supplementation of a nutraceutical combination consisted of berberine, red yeast rice, and policosanols during 4 weeks might cause decrease in HOMA-IR as an indicator of insulin resistance, while significant increase was obtained in QUICKI of hypercholesterolemic patients. Diminution of G:I ratio in our analysis is in agreement with findings from randomized trial conducted in T2DM patients showed that the *BV* extract for 3 months could improve IR (23). 

 The results of estimated relative treatment effect represented that *BV* juice caused apparently reducing effects on insulin and C-peptide levels consistent with the findings reported by Affuso* et al.* (22). In contrast, post-adjusted models showed significant increase in C-peptide levels in response to *BV* treatment, which is consistent with Yin *et al*. (24) findings attributed to remarkable increase in both fasting and postprandial C-peptide levels in patients treated with combinative effects of berberine and insulin therapy (T2DM patients). However, our findings based on the estimated relative treatment effect supported present hypothesis and suggested that *BV* supplementation could ameliorate HOMA-IR as an IR determinant. This is in accordance with the interventional evidence postulated for T2DM (15, 24). In line with our findings, Di-Pierro *et al*. (15), showed that a herbal extract contained *Berberis* *aristata* can lessen the mean level of HOMA-IR in T2DM patients. Taken together, *BV* supplementation seems to cause general controlling effects on glycemia in keeping with improvement on insulin secretion profile to possibly overcome the physiologic condition describing less insulin responsiveness. Additionally, Lu and coworkers found out that berberine treatment in diabetic rats could increase insulin level and preserving β-cell number in pancreas (20). Accordingly, to support the provided insights on the plausible benefits of *BV* fruit, the raised relative treatment effects observed in HOMA-B as an indicator of β-cell functionality suggested *BV* as an effective herb on enhancing HOMA-B in patients with BBD (20, 25). 

 The current study was associated with some limitations. Most importantly, the duration of consumption of *BV* juice was short and remains unclear whether the longer period of time may accompany with stronger evidence on insulin related indices. Also, another problem with this approach is that there was no similar controlled clinical trials published about BBD to be compared in results and almost all the clinical trials have tended to focus on the effects of *BV* on diabetic models.

## Conclusion

 This study suggested that *BV* juice caused regulatory roles on HOMA-IR and enhanced HOMA-B with the possible metabolic controlling effects on the growth promoting outcome of insulin. Taken together, our findings suggest that regular *BV* juice consumption might be effective on controlling insulin-related indices in benign breast disease. 

## Abbreviation

BBD, benign breast disease; *BV*, *berberis vulgaris*; BC, breast cancer; FBS, fasting blood sugar; G:I, glucose to insulin ratio; HOMA-IR, homeostasis model assessment; IR, insulin resistance; T2DM, Type 2 diabetes mellitus; OR, odds ratio; QUICKI, quantitative insulin sensitivity check index; WHO, world health organization; WLS, weighted least squares.
